# Cardiovascular health and cognitive function in autism and intellectual disability

**DOI:** 10.1192/j.eurpsy.2026.10347

**Published:** 2026-07-31

**Authors:** K. Krysta

**Affiliations:** Department of Rehabilitation Psychiatry, Medical University of Silesia, Katowice, Poland

## Abstract

**Abstract:**

Cardiovascular health plays a critical and often underappreciated role in shaping cognitive function across the lifespan in individuals with autism spectrum disorder (ASD) and intellectual disability (ID). From early development onward, the heart–brain connection exerts a powerful influence on neurocognitive trajectories. Children born with congenital heart defects are at increased risk of neurodevelopmental delays, including ASD and ID, partly due to impaired cerebral oxygenation during critical periods of brain maturation. These effects may be further amplified by shared genetic pathways that influence both cardiac development and neural connectivity, leading to long-term alterations in cognition, attention, and executive functioning.

As individuals with ASD/ID transition into adulthood, cardiovascular vulnerability often increases rather than diminishes. Adults in this population are more likely to develop cardiovascular diseases at a younger age compared with the general population, driven by a combination of biological susceptibility, chronic low-grade inflammation, metabolic dysregulation, and lifestyle-related risk factors such as physical inactivity, obesity, and medication effects. These cardiovascular conditions can accelerate cognitive decline through mechanisms including chronic cerebral hypoperfusion, endothelial dysfunction, and neuroinflammatory processes, all of which negatively affect brain structure and function.

Importantly, cognitive changes related to cardiovascular health in ASD/ID may remain underrecognized, as they are frequently attributed solely to neurodevelopmental factors rather than to potentially modifiable somatic conditions. This highlights the need for a lifespan-oriented model of care. Early neurodevelopmental surveillance in children with cardiac conditions should be integrated with routine cognitive and behavioral monitoring. In adulthood, proactive cardiovascular screening, risk reduction strategies, and individualized lifestyle interventions should become standard components of care.

**Disclosure of Interest:**

None Declared

